# Observation of ultra-large Rabi splitting in the plasmon-exciton polaritons at room temperature

**DOI:** 10.1515/nanoph-2023-0162

**Published:** 2023-07-10

**Authors:** Min Zhang, Yuan Tian, Xingzhou Chen, Zheng Sun, Xiaolong Zhu, Jian Wu

**Affiliations:** State Key Laboratory of Precision Spectroscopy, East China Normal University, Shanghai, 200241, China; Collaborative Innovation Center of Extreme Optics, Shanxi University, Taiyuan, Shanxi 030006, China; Chongqing Key Laboratory of Precision Optics, Chongqing Institute of East China Normal University, Chongqing 401121, China; CAS Center for Excellence in Ultra-intense Laser Science, Shanghai 201800, China

**Keywords:** metasurface, plasmon-exciton polariton, Rabi splitting

## Abstract

Modifying the light–matter interactions in the plasmonic structures and the two-dimensional (2D) materials not only advances the deeper understanding of the fundamental studies of many-body physics but also provides the opportunities for exploration of novel 2D plasmonic polaritonic devices. Here, we report the plasmon-exciton coupling in the hybrid system with a plasmonic metasurface which can confine the electric field in an extremely compact mode volume. Because of the 2D feature of the designed and fabricated Al plasmonic metasurface, the confined electronic field is distributed in the plane with the same orientation as that of the exciton dipole moment in the transition metal dichalcogenides monolayers. By finely tuning the geometric size of the plasmonic nanostructures, we can significantly modify the dispersion relation of the coupled plasmon and the exciton. Our system shows a strong coupling behavior with an achieved Rabi splitting up to ∼200 meV at room temperature, in ambient conditions. The effective tailoring of the plasmon-exciton coupling with the plasmonic metasurfaces provides the testing platform for studying the quantum electromagnetics at the subwavelength scale as well as exploring plasmonic polariton Bose–Einstein condensation at room temperature.

## 1 Introduction

Plasmon-exciton coupling is an important phenomenon in nanophotonics where light interacts with matter. Thanks to localized surface plasmon resonances, light can be confined in the nanoscale level, which leads to a substantial increase of the electromagnetic field, thus uncovering the remarkable strong light–matter interactions. Through the strong coupling, the plasmon polaritons can be formalized, which are the driven collective oscillation of electrons by light, providing the foundation for various applications ranging from coloration, opt-communication, and photocatalysis to biological sensing [1–5]. Owing to the two-dimensional (2D) feature of the excitations, plasmon polaritons supported by 2D metallic metasurfaces are of particular interest. Tight mode confinement and remarkable electrostatic tunability of plasmonic metasurfaces lead to new platforms for the investigation of light–matter interactions.

Recently, the TMDCs have received considerable attention owing to their promising applications in optoelectronics and electronics [6–9]. Thus, they are widely explored as candidates for integration with solids. In addition, monolayer TMDCs possess extraordinary oscillator strength and considerable excitonic binding energy (0.3–0.5 eV) [10, 11], which result from the significant Coulomb interaction and reduced dielectric screening in atomically thin structures. Therefore, integrating the monolayers in an optical resonator can expect to intrigue the intense energy exchange between the electromagnetic field of the light and the excitons [12–16].

Combining the two-dimensional van der Waals crystals with the plasmons in the metal can now be achieved with the plasmonic nanocavities with a gap width down to a subwavelength scale. However, the reported strong coupling of the 2D-based plasmon-exciton system has exploited the nanocavities formed through randomly spraying the single nanowires or nanoparticles over the monolayers [17–22]. Hence it is unable to systematically control and tune the coupling. Moreover, the usual vertical nano-resonator is hard to couple with the 2D materials since the electromagnetic field and the dipole moment is not aligned in the same manner. Therefore, large Rabi-splitting cannot be achieved in such nanostructures and would hinder the efficient energy transferring between the plasmons and excitons, thus lowering the performance of the devices.

In this work, we demonstrate a 2D hybrid coupling system showing an ultra-large Rabi splitting up to 200 meV. It is realized by plasmonic arrays and a monolayer WS_2_. The pillars can significantly confine the electromagnetic field in the 2D plane with the same orientation of the dipole moment of the TMDCs monolayers. In addition, the employment of the monolayer WS_2_ with a high refractive index can further encapsulate the electromagnetic field in the *xy*-plane. By modifying the diameters (*D*) and the periods (Γ), the energy splitting and the number of excitons involved in the coupling can be finely changed. Finally, the splitting feature is presented by performing the photoluminescence measurements, which unambiguously show the hybridization of the modes for the light and the matter interaction. The sufficient modification of the exciton properties with plasmons can raise the exploration of applications such as the development of optoelectronic devices, optical switching, and sensing [23–25].

**Figure 1:**
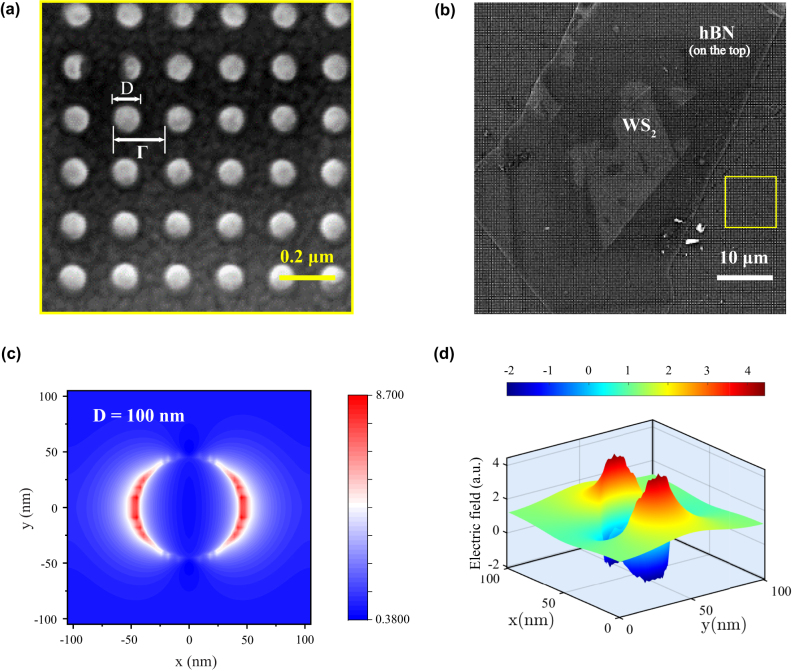
Optical image and E-field distribution of the hybrid system. (a) Larger magnification of SEM images of the bare plasmonic metasurface with a typical diameter *D* ∼ 100 nm and a period Γ ∼ 200 nm. (b) Lower magnification SEM image of the plasmonic metasurface with a transferred monolayer WS_2_ capped with a thin film hBN on top. The typical size of the monolayer is about 20 μm × 20 μm. The yellow square indicates the zoomed-in area. (c) The calculated 2D electric-field intensity distribution for the bare plasmonic metasurface in top–down view *xy*-plane. The diameter of the single nano-pillar is 100 nm. (d) The calculated 3D electric-field distribution for the plasmonic metasurface with monolayer WS_2_ for the same pillar size shows a typical dipolar field distribution at the rim of the disk.

The fabricated plasmonic metasurface is characterized by SEM in Figure 1(a), which shows that the structure is composed of regular cylindrical arrays. The metasurface is designed with the top Al disks array, whose diameter varies from 90 nm to 130 nm, and the thickness is 20 nm. The middle layer is composed of polymeric pillars with a refractive index of about 1.5, and the diameter varies from 90 nm to 130 nm, the same as the disk array, whose height is 35 nm. The thickness of the bottom Al film is 20 nm as well (see the detailed fabrication procedure in Methods). The whole structure is established on a SiO_2_ (glass) substrate. Monolayer WS_2_ and thin film h-BN are mechanically exfoliated from the bulk onto PDMS and transferred to silicon and metasurface using a standard dry transfer process [26, 27]. The hybrid system is illustrated in Figure 1(b). The transferred WS_2_ is then identified by photo imaging and PL measurements at room temperature. The distribution of the simulated electric field at the resonance wavelength (∼650 nm for *D* = 100 nm) in the *xy*-plane is shown in Figure 1(c) (the electric field in the *xz*-plane can be referred to the Supplementary material in Note 5). We can find that the electric field is symmetrically distributed on both sides of the cylinder, the same as in a dipolar manner. Thus, when the monolayer is placed on the top edge of cylinders, excitons in two-dimensional materials are likely to interact with electric dipoles excited by the plasmonic nanostructures. It is worth noting that, unlike the plasmonic cavities formed by particle-WS_2_-metals [28–31], the plasmonic metasurface used here with an open architecture has unique advantages for experimental detection and potential applications in the display industry. Figure 1(d) presents the simulated (finite-difference time-domain (FDTD) full wave simulation) electric field distribution of Al nano-disk with WS_2_ monolayer, we can see that by applying WS_2_ on the surface, the electric field at the top interface of the metasurface-WS_2_ hybrid structure can be significantly enhanced. It is worth mentioning that the applied hybrid plasmonic metasurface with Al holes array under the Al disks ensures the optical field is mainly localized in the top layer which to the maximum extent overlaps with the transferred WS_2_ monolayer.

**Figure 2:**
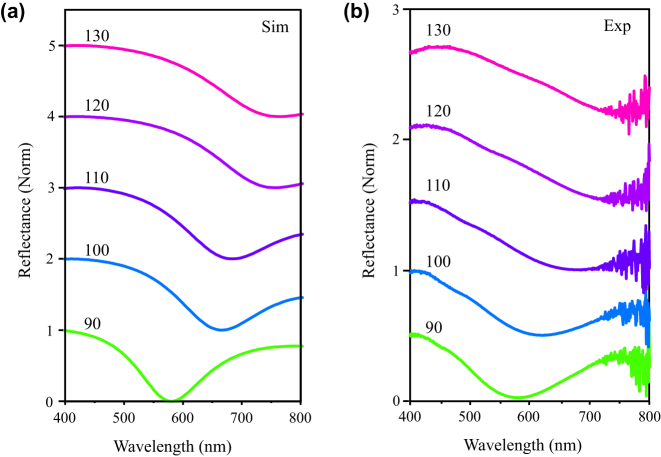
The simulated reflectivity spectra versus the experimental reflectivity spectra. (a) The simulated reflectivity spectra for the bare plasmonic metasurface with various diameters from 90 nm up to 130 nm. The plasmon mode blue shifts with the increased size. (b) The corresponding experimental reflectivity spectra. The intensity noise above 750 nm can be observed because of the incapability of the white source in that regime.

To resonantly couple to the monolayer WS_2_, we designed the nanostructure with the plasmon modes covering the exciton energy of the WS_2_ monolayer, which is at 610 nm. We use the FDTD method to calculate the optical properties of the metasurface. As shown in Figure 2(a), the dip in the simulated reflectivity spectra with cylinders of different (increasing) size redshifts. Before investigating the hybrid system, we primarily characterized the plasmonic metasurface in Figure 2(b), which shows the measured reflectivity spectra. We can find that the simulation results are in good agreement with the experimental ones. Note that the experimental spectra are broader than the spectra in the simulation because of the defects introduced during preparation, which may cause extra damping in the hybrid system. From Figure 2(b), the reflectivity spectrum exhibits a dip close to 580 nm with broadband when the diameter is about 90 nm. There is a dip near 600 nm when the diameter of the disk array is about 100 nm. The plasmonic resonances are sensitive to the geometrical aspect ratio, which can be attributed to the change in the average electron density [32]. In our work, by varying the diameter of the cylinders, the surface plasmon resonance can be tuned, which allows the plasmon mode to match with the ex_A_ of the WS_2_ monolayer.

In addition, considerable coupling strength and low damping loss are those two indispensable aspects for realizing efficient coupling. In a strong coupling plasmon-exciton system, the existence of a splitting in frequency implies the coherent exchange of energy between the SPP field [33] and the oscillators (emitters). The strong coupling regime can be defined as the splitting being large enough compared to the linewidths of the coupled states. The optical mode splitting with the energy separation between the normal modes can be also called the Rabi splitting [34], originally derived from fully quantum theory. The mode-splitting is [35]

$$\Omega_{R}\propto d\sqrt{\frac{\omega_{0}N}{\hbar \varepsilon_{0}V}}$$

where *d* is the dipole moment of a two-level system, *ω*_0_ is the frequency at the resonance, *N* is the number of emitters [36] and *V* is the mode volume within a plasmonic cavity. To enhance the coupling strength *g*(∝Ω_R_), one of the methods is to decrease the mode volume *V*. Excited plasmon polaritons of plasmonic metasurfaces can effectively confine the electromagnetic field in the subwavelength scale below the diffraction limit. Thus, it can provide a compact and stable environment for strong coupling at room temperature. When the rate of coherent energy transfer between an excitonic transition and plasmons is faster than their average dissipation rate, the system can undergo a strong coupling regime, forming so-called plexcitons. The interaction between excitons and the plasmon modes can be continuously tuned to reach different coupling strengths by adjusting the dimensions of the nanostructure, for example, nanoparticle size, gap thickness, and shape.

**Figure 3:**
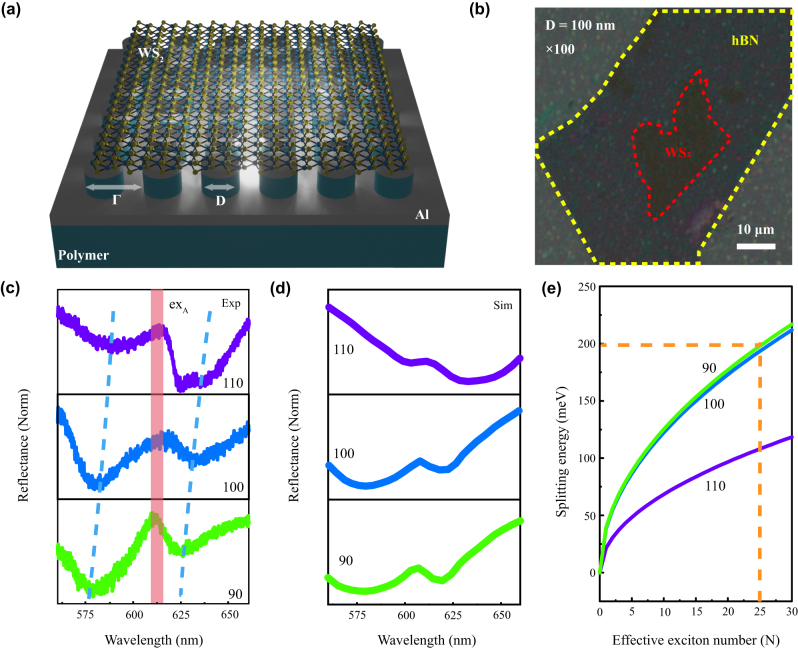
The reflectivity spectra in the coupled hybrid system with different sample sizes. (a) Schematic diagram of the sample structure where on the top layer is the monolayer WS_2_ and beneath it is the metasurface. (b) Optical microscope image of the sample. WS_2_ monolayer and hBN film are illustrated with the red dotted and yellow dotted lines respectively (the monolayer WS_2_ is capped with a thin film hBN). (c) The diameter dependence differential reflection spectra. It shows two well-defined plasmon-polariton states at both sides of the ex_A_ energy. The vertical red line represents the WS_2_ ex_A_ energy, and the blue dotted curves trace the dispersion of plasmon-polariton modes. (d) FDTD-simulated reflection spectra for the samples. (e) The splitting energy (see the Supplementary material Note 1 for more details) versus the effective exciton number *N*. The structure sizes are for *D* = 90 nm, *D* = 100 nm, and *D* = 110 nm.

Figure 3(a) presents the sketched structure of the plasmonic metasurface combined with a WS_2_ monolayer, where the plasmonic substrate is formed by a 20 nm-thick Al film sputtered onto the substrate with dielectric pillars. The top structure is similar to the bottom, especially its shape is cylindrical and it is arranged periodically according to a period of 200 nm. Figure 3(b) shows a typical optical image of the hybrid structures under a 100× microscope objective, in which the red dots enclosed are WS_2_ monolayer and the yellow dots enclosed are h-BN thin film. Here, by adjusting the diameter in lithography, we can achieve feasible tuning of plasmonic resonances supported in the system. Figure 3(c) illustrates the measured differential reflectivity. It should be noted that the demonstrated samples here are with plasmonic resonances which are located near the critical crossing point of the Rabi-type mode splitting. It is implemented micro-zone by an aperture to a 2 μm spot. The differential reflectivity is calculated as $\frac{\Delta R}{R}=\frac{I_{\text{bg}}-I_{\text{sample}}}{I_{\text{bg}}}$ , where *I*_bg_ is the reflectance intensity of the background area with bare structure, and *I*_sample_ is the reflectance intensity of the WS_2_ monolayer on the metasurface. The two separate valleys in the white light-illuminated reflectivity spectra indicate the plasmon and exciton in the hybrid system are strongly coupled.

Noted that, when the sample diameter is set to be 100 nm (close to the critical splitting point), the energy difference between the upper and lower energy branches is about 200 meV, which shows a remarkable Rabi-type mode splitting and indicates the strong interaction between the in-plane excitons and plasmons. By using the equation $\hbar \Omega_{\text{R}}=2\sqrt{g^{2}-1/4{\left(\hbar \gamma_{\text{ex}}-\hbar \gamma_{\text{pl}}\right)}^{2}}$ (Ω_R_ is the Rabi splitting, *g* is the coupling strength), and the experimental halfwidths for plasmon mode *ℏγ*_pl_ = 176 meV and the exciton *ℏγ*_ex_ = 34 meV (see the Supplementary material Note 2 for more details), we can derive the coupling strength *g* ∼ 123 meV. This result satisfies the inequality $g>\left|\hbar \gamma_{\text{ex}}-\hbar \gamma_{\text{pl}}\right|/$ 2, a necessary condition for the formation of strongly coupled plexciton states [37]. Figure 3(d) presents the according simulation, which shows coupled mode interference [38]. An obvious mode splitting is presented on both sides of the exciton position (610 nm, denoted with a pink vertical bar in Figure 3(c)), further confirming the strong coupling behavior. For localized dipole plasmon resonances in disks, the effective wavevector *k* for the dipole mode is simply given by *k* ∼ 2/*D*, where *D* is the diameter of the disks. It should be noted that, for small wavevector *k*, the coupled plasmon–exciton dispersion has a negligible deviation from the simple $\sqrt{k}$ dispersion [39]. In Figure 3(e), we plot the splitting energy as a function of the effective number *N* for three conditions (*D* = 90 nm, 100 nm, and 110 nm). Note 1 in the Supporting Information shows the details of the function, (see Supporting Information). We simulated the mode volume (*V*) and the factor number *F* using a numerical method, FDTD. The Rabi splitting ( $\Omega_{\text{R}}\left.\right)$ is obtained in measurement (in Figure 3(c)). In the same effective number *N*, it clearly shows that the splitting energies of 90 nm and 100 nm samples are similar, both stronger than in the case of 110 nm. It indicates that the *N* values can be changed by modulating the diameters of the structures. It should be mentioned that although we design the disks array, which is arranged in a periodic architecture, we mainly utilize the localized plasmon mode to investigate its coupling with ex_A_ of WS_2_. We obtain the strong coupling dispersion by varying the diameters of the disks. As the mode we used originated from individual disk, not from the lattice mode involving all the disks, we calculated the mode volume within a unit cell of a disk for the localized plasmonic mode [30, 40, 41]. Hereby, our work provides a new approach for the strong coupling of plasmonic structures with TMDCs and promises to achieve the strong coupling of a few excitons to the plasmon.

The measured splitting in the reflectivity is a bit larger than that in the simulated spectra. This can be attributed to the simplicity of the simulation model exploited to predict the reflectivity spectra. In particular, the modeled reflectivity spectra correspond to the energy that is coupled from the emitter into the plasmon and subsequently radiated from the plasmon into free space with an ideal case where WS_2_ is rigidly suspended on top of the Al disks; the measured spectra may also include contributions from light at the side walls of the Al disks covered by the WS_2_ monolayer.

**Figure 4:**
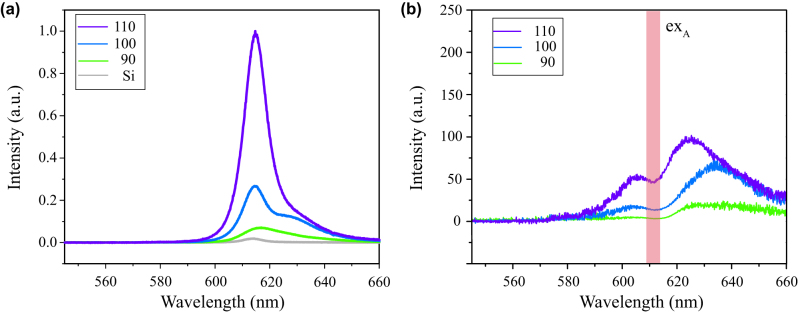
PL spectra in the coupled hybrid system with different sizes. (a) The measured PL spectra for the samples with *D* = 90 nm, *D* = 100 nm, and *D* = 110 nm. The grey line is the PL for the WS_2_ monolayer on a SiO_2_/Si substrate as a contrast. (b) PL enhancement spectra for the three different diameters divided by that of the control sample. It shows two well-defined plasmon-polariton states at both sides of the ex_A_ energy (red line).

To further confirm the strong coupling in the hybrid system, we carried out photoluminescence measurements. The samples were pumped by a continuous wave laser (532 nm). The pump beam was focused by a reflecting microscope objective (0.5 numerical aperture, N.A.) to a spot size of 1 μm on the sample. The emission was collected by the same microscope objective and analyzed with a spectrometer equipped with a charged couple device (CCD). Figure 4(a) shows the measured PL spectroscopy collected under the same experimental conditions on the different samples at room temperature. Comparing the WS_2_ monolayer on Si with the one on the metasurface, a slight red shift of the PL emission peak can be observed, due to the stress introduced during the transfer process [42]. With the increasing diameter, the peak of PL increases as well. When the diameter of a sample reaches 110 nm, the PL enhances 53-fold compared to the PL of the sample on the Si wafer [43]. The enhancement of spontaneous emission rates in an optical resonant cavity, known as the Purcell effect, can be estimated by the Purcell factor. For a certain cavity, the Purcell factor $F_{\text{p}}\propto \frac{Q}{V}$ , where *Q* and *V* are the quality factor and mode volume of the plasmonic cavity, respectively [44]. The way to increase the Purcell factor is again to reduce the mode volume [45]. Therefore, the electric field confinement becomes more obvious for the PL enhancement while the diameter becomes bigger under certain conditions. And it opens up new routes for future applications that can be used for sensitive measurements. Here the result is also in line with the simulation results.

Figure 4(b) demonstrates the PL enhancement ratio by dividing the values of the different samples by the values on the Si wafer. As in Figure 4(b), we can find that the PL spectra show two different prominent peaks (*ω*_+_ and *ω*_−_) resulting from the strong interaction between plasmon and emitters [46], which match with the reflectivity spectra. The dips in the spectra are almost in the same position, which is the narrow-band exciton of WS_2_. It is obviously shown that the up branch (higher energy) displays a stronger signal than the lower branch (lower energy). When the diameter of the cylinder array is set to 100 nm, the plasmon-excitonic PL mode-splitting is up to 112 meV. It should be mentioned that the observed splitting in the PL enhancement spectrum is smaller than the splitting (200 meV) in the reflectivity spectra. The difference of spectral Rabi splitting in different measured spectra is mostly because they contain different contributions from the plasmon or exciton channel [47] and the plasmon channel usually has much larger splitting than the exciton channel because of its larger dipole moment [48]. PL behaviors originate from bounced channels with the complicated interplay among plasmons, phonons, and excitons, resulting in a degraded mode splitting strength. As the diameter increases, the lower branch of PL becomes clear and shows a blue shift. It further indicates that our designed structure obtaining the field localization is critical to minimizing mode volume and achieving strong coupling.

In summary, we have demonstrated a strong plasmon-exciton coupling between the plasmonic metasurface and the WS_2_ monolayer. Strong plasmon-exciton coupling is studied by the differential reflectivity measurements, PL measurements and FDTD simulations. We present a giant tunable Rabi splitting and a small number of involved coupling excitons by changing the diameter of the nano-arrays. Combining the distinct properties of localized surface plasmon resonances and the unique properties of 2D semiconductors such as their exceptional exciton binding energies and atomic thickness, the possibility of revealing the plasmon-exciton condensation is an interesting feature offered by such an “open cavity”.

## 2 Methods

### 2.1 Fabrication procedure of the Al metasurface

A silicon mold was fabricated by using electron-beam lithography (EBL, JEOL JBX 100 keV) and dry etching. EBL with a focused Gaussian beam was used to define shapes. The sample was fabricated on a 0.5 mm thick, 4-inch glass wafer. The column structure is reproduced into the Ormocomp layer by room temperature nanoimprint using a silicon stamp with an anti-adhesion coating. The Ormocomp film was cured by exposure to UV light and separated from the silicon master, which is subsequently stripped off the glass substrate. Then, at the pressure of 10^−6^–10^−5^ mbar, a 20 nm Al film was deposited by an electron beam evaporator (Alcatel SCM 600) at 5 Å/s. About polymer-coated samples, a sufficiently thick layer of PMMA (10 % 950 PMMA in Anisole, Micro Chem Corp) was spin-coated (500 rpm) on top to avoid Fabry–Pérot interference. To avoid the affection of the air, in our experiments, we performed multiple ways to decrease the degradation caused by the passivation, 1. The samples are carefully stored in a nitrogen-filled environment; 2. A thin film hBN was transferred to cover the effective area to protect the WS_2_ monolayer and the passivation of the Al nano-disk metasurface [49, 50].

### 2.2 Descriptions of strong coupling for the hybrid system

Mode splitting can be interpreted by the Jaynes–Cummings Hamiltonian function. We can calculate the *ω*_±_, which yields the eigenvalue problem for the hybrid state (two modes system).

$$\left(\begin{array}{cc} \frac{i\gamma_{\text{pl}}}{2}+\omega_{\text{pl}} & g\\ g & \frac{i\gamma_{\text{ex}}}{2}+\omega_{\text{ex}} \end{array}\right)\left(\begin{array}{c} \alpha \\ \beta \end{array}\right)=\omega_{\pm }\left(\begin{array}{c} \alpha \\ \beta \end{array}\right)$$

*ω*_pl_ and *ω*_ex_ as the Al disk plasmonic mode and WS_2_ exciton frequencies, *γ*_pl_ and *γ*_ex_ are the loss of Al plasmonic resonance linewidth and A exciton linewidth in WS_2_. Using this function, modes can be changed as

$$\omega_{\pm }=\frac{1}{2}\left(\omega_{\text{pl}}+\omega_{\text{ex}}\right)\pm \sqrt{g^{2}+\frac{\delta^{2}}{4}}$$

In this function, *δ* = *ω*_pl_ − *ω*_ex_, use simulation detuning data Al plasmon mode and A excitons frequency. With the increasing diameter, the coupling strength also changes.

As a result, the plasmon-exciton coupling is confined within the *xy*-plane. Thus, the exciton numbers *N* can be mathematically expressed as [51]:

$$g=\sqrt{\frac{\omega }{\hbar \varepsilon_{0}Re\left[\frac{d\left(\omega \varepsilon \right)}{d\omega }\right]V}\cdot N\cdot F}\cdot d_{0}$$

Which *N* is the effective exciton numbers in the system, *d*_0_ = 56 Debyes = 1.848 × 10^−28^ C m. *V* is the mode volume. **F** reveals the integrated plasmon-exciton coupling efficiency toward different orientations at different positions

In this function, we note that *g* relates to *V*, *N*, and **F** **.** By modulating the size of the Al disk, we can tune *V* and **F** accordingly. By using the simulated E-field distributions, we can calculate the mode volume easily. Noted that the other parameters in this equation are constants for a certain plasmonic cavity. It should be mentioned that, considering the naturally polarized properties of the excitation laser, the dipolar distribution of the E-field will result in a symmetric diagram with two half-moon-shaped E-field distributions. To be excited as, for example, quantum light sources, it typically should only employ one of the excited edges as the light source, thus the used mode volume for the calculations in our case is halved from the entire cavity of a unit cell of the periodical structure. The square root relation of the *g* versus *N* can be thus plotted with the calculated mode volumes *V* for the varied diameters *D*.

The *d*_0_ will only be influenced by the parameters of the materials, while *N* is difficult to change by regular manipulations because excitons are normally not localized in the usual system. But it is noted that the excitons in WS_2_ monolayers are completely in-plane in our system, which makes different coupling manners in different diameters. We plot the coupling strength as a function of the effective exciton number for *d* = 90 nm, 100 nm, and 110 nm in Figure 3e, respectively. When we increase the disk’s diameter, the average coupling strength tunes to be smaller.

## Supplementary Material

Supplementary Material Details
