# Supplementary material for: Observation of ultra-large Rabi splitting in the plasmon-exciton polaritons at room temperature
Source: Nanophotonics. 2023 Jul 10;12(16):3267–75. doi: 10.1515/nanoph-2023-0162 (PMC11501516; doi:10.1515/nanoph-2023-0162)
Supplement: Supplementary file 1 — Supplementary Material Details [file j_nanoph-2023-0162_suppl_001.docx]

**Observation of ultra-large Rabi splitting in the plasmon-exciton polaritons at room temperature**

Min Zhang^1*^, Yuan Tian^1*^, Xingzhou Chen^1^, Zheng Sun^1#,2^, Xiaolong Zhu^1#,2^, Jian Wu^1#,2,3,4^

*^1^State Key Laboratory of Precision Spectroscopy, East China Normal University, Shanghai, 200241, China*

*^2^Collaborative Innovation Center of Extreme Optics, Shanxi University, Taiyuan, Shanxi 030006, China*

*^3^Chongqing Key Laboratory of Precision Optics, Chongqing Institute of East China Normal University, Chongqing 401121, China*

*^4^CAS Center for Excellence in Ultra-intense Laser Science, Shanghai 201800, China*

**^*^These authors contributed equally to this work**

^#^Email: [zsun@lps.ecnu.edu.cn](mailto:zsun@lps.ecnu.edu.cn); xlzhu@lps.ecnu.edu.cn; [jwu@phy.ecnu.edu.cn](mailto:jwu@phy.ecnu.edu.cn);

**Note 1 Derivation of** $\boldsymbol{N}$ **from plasmon-exciton coupling.**

The number of plasmon-exciton coupling was an important discussion in this part.

$u\left( r \right)= \frac{1}{2}\varepsilon_{0}Re\left[ \frac{d\left( \omega\varepsilon\right)}{d\omega} \right]\left\langle{E\left( r,t \right)E\left( r,t \right)}^{*} \right\rangle+\frac{1}{2}\mu_{0}Re\left[ \frac{d\left( \omega\mu\right)}{d\omega} \right]\left\langle{H\left( r,t \right)H\left( r,t \right)}^{*} \right\rangle$ (S1)

$\varepsilon_{0}$ is the free-space permittivity,$\mu_{0}$ is the free-space permeability,$\varepsilon$is the dielectric constant,$\mu$ is the relative permeability,$\mathrm{Re}\left[ \frac{d\left( \omega\varepsilon\right)}{d\omega} \right]$ stands for the value of the real part of $\left[ \frac{d\left( \omega\varepsilon\right)}{d\omega} \right]$ and $\mathrm{Re}\left[ \frac{d\left( \omega\mu\right)}{d\omega} \right]$ stands for the value of the real part of $\left[ \frac{d\left( \omega\mu\right)}{d\omega} \right]$

$E\left( r,t \right)= E\left( r \right)e^{-i\omega t}+E^{*}\left( r \right)e^{-i\omega t}$ (S2)

$u\left( r \right)= \varepsilon_{0}Re\left[ \frac{d\left( \omega\epsilon\right)}{d\omega} \right]\left\langle{E\left( r \right)E\left( r \right)}^{*} \right\rangle+\mu_{0}Re\left[ \frac{d\left( \omega\mu\right)}{d\omega} \right]\left\langle{H\left( r \right)H\left( r \right)}^{*} \right\rangle$ (S3)

the mode volume $V$ can be calculated by

*V*$=\frac{\int u\left( r \right)dr}{Max(u\left( r \right))}$ (S4)

In this system, it can be seen that

$\varepsilon_{0}Re\left[ \frac{d\left( \omega\epsilon\right)}{d\omega} \right]\left\langle{E\left( r \right)E\left( r \right)}^{*} \right\rangle\gg\mu_{0}Re\left[ \frac{d\left( \omega\mu\right)}{d\omega} \right]\left\langle{H\left( r \right)H\left( r \right)}^{*} \right\rangle$ (S5)

Coupling strength of an ensemble of excitons.

Two dimensions of excitons only spread in WS_2_, which have continued in the distribution in space. In our experiment, the coupling strength $g$^1^.

$g=\sqrt{\frac{ћ\omega\cdot N}{\epsilon_{0}Re\left[ \frac{d\left( \omega\varepsilon\right)}{d\omega} \right]V}}d_{0}\cdot\left| F_{\mid\mid}\left( r \right) \right|/ћ$ (S6)

$g$ is the coupling strength between the exciton and the cavity (plasmon) mode at the position, $d_{0}$ = 56 Debye≈1.848×10^-28^ C·m. $\left| F_{\mid\mid}\left( r \right) \right|= \sqrt{\mathbf{F}}$ being parallel to the 2D-material plane.

The factor $\mathbf{F}$ numerically by using

$\mathbf{F}=\frac{\sum_{i} \left( E_{x,i}^{2}+E_{y,i}^{2} \right)}{\sum_{i} \left( E_{x,i}^{2}+E_{y,i}^{2}+E_{z,i}^{2} \right)}$ (S7)

$g=\sqrt{\frac{\omega}{ћ\epsilon_{0}Re\left[ \frac{d\left( \omega\varepsilon\right)}{d\omega} \right]V}\cdot N\cdot\mathbf{F}}\cdot d_{0}$ (S8)

**Note 2 The differential reflectivity spectrum of monolayer WS_2_ on the SiO_2_/Si.**


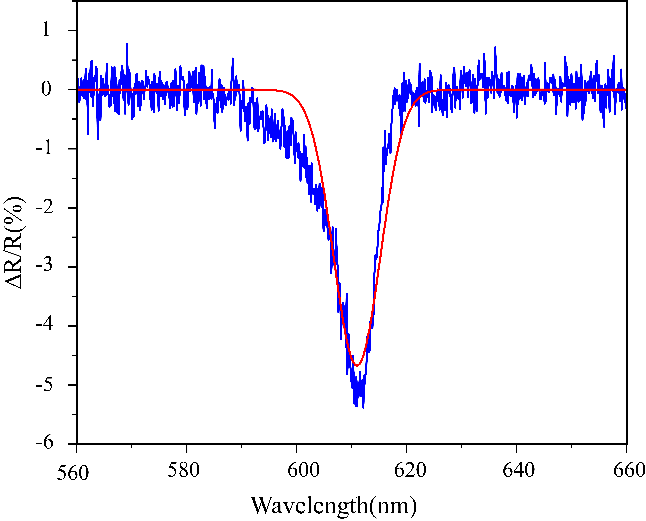


Figure S1. The differential reflectivity spectrum of monolayer WS_2_ on the SiO_2_/Si.

$$\frac{\Delta R}{R}= \frac{I_{bg}-I_{sample}}{I_{bg}}$$

As Figure S1 shows, the blue line is obtained using differential reflection, and the red line is the result of Gaussian fitting for the blue line. The position of the dip is 610 nm, and the FWHM is 10nm, so the exciton $\hbar\gamma_{ex}=34$ meV.

**Note 3 The structure color of the metasurface for different sizes.**


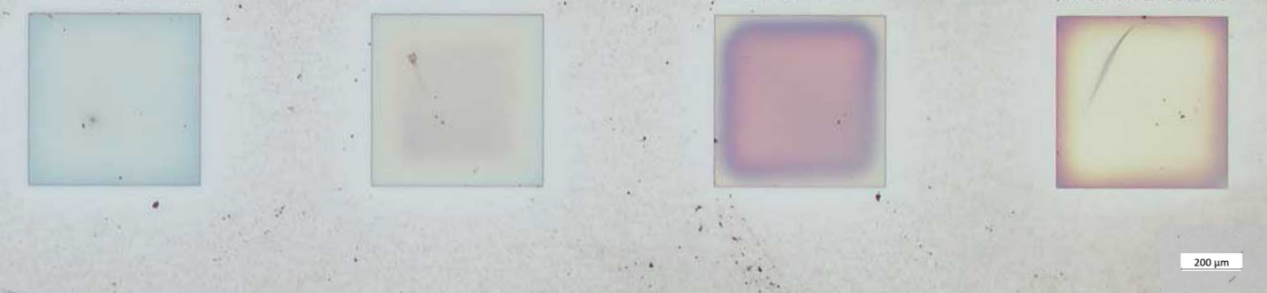


Figure S2. The structure colors.

**Note 4 Reflectance spectra of the structure.**


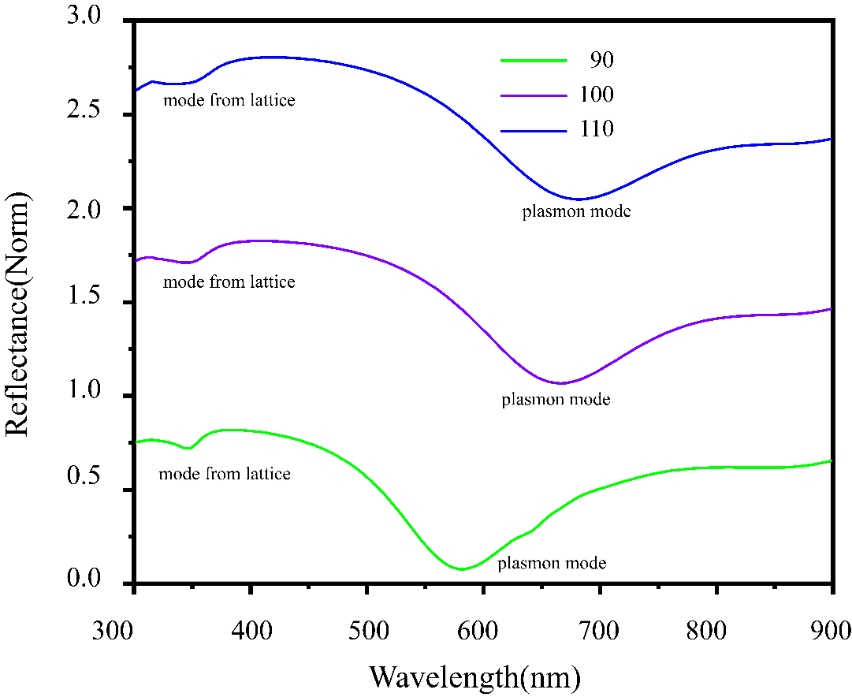


Figure S3. The simulated reflectivity spectra of the sample.

The first dip is lattice mode of the Al nano-disk meta-surface. The second dip is plasmon mode of the single Al nano-disk. Lattice mode is around 350 nm, so the dip in the reflection spectrum is attributed to the localized plasmon mode of the individual Al nano-disks. As the diameter increases, the lattice mode does not change, but the plasma mode tends to be redshifted.

**Note 5 Electric field intensity distributions comparison for the structures with and without the polymeric pillar arrays.**


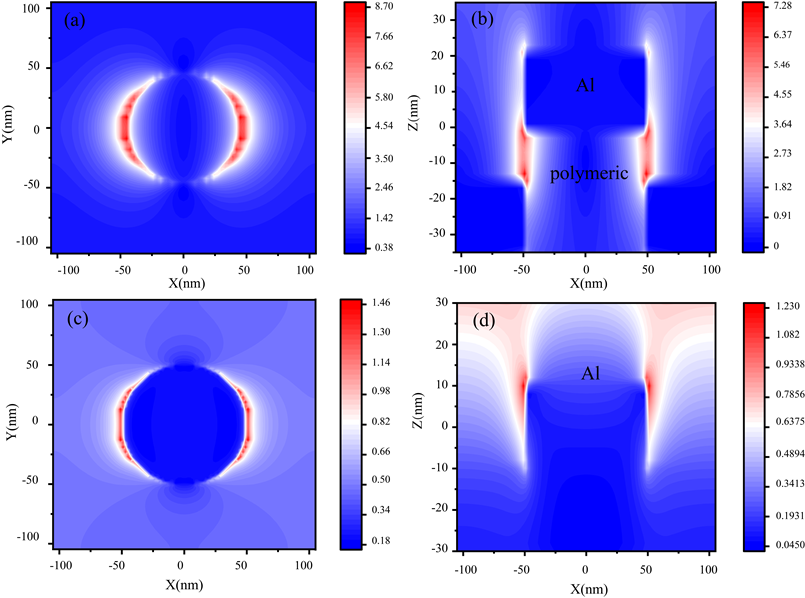


Figure S4. The calculated 2D electric field intensity distributions for plasmonic metasurface with and without the polymetric pillar arrays. (a) and (c), xy-plane. (b) and (d), xz-plane.

We simulated the structures as shown in Figure S4. It presents the electric field intensity distributions in xz plane and xz-plane of the sample (D = 100 nm) with and without the polymetric pillar arrays. Our simulations show that the pillar arrays can help to enhance the electric field and localize it within a small space. Also, the distribution of the electric field in the E_xz_ shows the divergence for the sample without the polymetric pillar arrays.

**Reference:**

(1) Qin, J.; Chen, Y. H.; Zhang, Z.; Zhang, Y.; Blaikie, R. J.; Ding, B.; Qiu, M. Revealing Strong Plasmon-Exciton Coupling between Nanogap Resonators and Two-Dimensional Semiconductors at Ambient Conditions. *Phys. Rev. Lett.* **2020**, *124* (6), 63902.
